# Case Report: Discordance between RNA-identified *ETV6::RET* and clinically reportable DNA profiling in salivary gland secretory carcinoma

**DOI:** 10.3389/fonc.2026.1967793

**Published:** 2026-09-24

**Authors:** Shogen Boku, Takao Fujisawa, Tomofumi Sakagami, Shigenori Kadowaki, Hironaga Satake, Hisateru Yasui, Koushiro Ohtsubo, Yasushi Shimizu, Tomoyuki Otsuka, Bunya Kuze, Riu Yamashita, Taro Shibuki, Yoshiaki Nakamura, Hideaki Bando, Takayuki Yoshino, Milan Radovich, Masao Yagi, Susumu Okano

**Affiliations:** 1 Department of Clinical Oncology, Kansai Medical University Hospital, Hirakata, Osaka, Japan; 2 Department of the Promotion of Drug and Diagnostic Development, National Cancer Center Hospital East, Kashiwa, Chiba, Japan; 3 Department of Head and Neck Medical Oncology, National Cancer Center Hospital East, Kashiwa, Chiba, Japan; 4 Department of Otorhinolaryngology, Head and Neck Surgery, Kansai Medical University, Hirakata, Japan; 5 Department of Clinical Oncology, Aichi Cancer Center Hospital, Nagoya, Aichi, Japan; 6 Department of Medical Oncology, Kochi Medical School, Kochi University, Nankoku, Kochi, Japan; 7 Department of Medical Oncology, Kobe City Medical Center General Hospital, Kobe, Hyogo, Japan; 8 Department of Medical Oncology, Kanazawa University Hospital, Kanazawa, Ishikawa, Japan; 9 Department of Medical Oncology, Hokkaido University Hospital, Sapporo, Hokkaido, Japan; 10 Department of Medical Oncology, Osaka International Cancer Institute, Osaka, Japan; 11 Department of Head and Neck Surgery, Central Japan International Medical Center, Minokamo, Gifu, Japan; 12 Division of Translational Informatics, Exploratory Oncology Research and Clinical Trial Center, National Cancer Center, Kashiwa, Chiba, Japan; 13 Department of Hepatobiliary and Pancreatic Oncology, National Cancer Center Hospital East, Kashiwa, Chiba, Japan; 14 Department of Gastrointestinal Oncology, National Cancer Center Hospital East, Kashiwa, Chiba, Japan; 15 Caris Life Sciences, Phoenix, AZ, United States; 16 Department of Otorhinolaryngology, The Jikei University School of Medicine, Minato-ku, Tokyo, Japan

**Keywords:** case report, comprehensive genomic profiling, DNA profiling, ETV6::RET, RNA sequencing, salivary gland secretory carcinoma

## Abstract

Secretory carcinoma of the salivary gland is a rare, fusion-driven malignancy in which kinase fusions can serve as therapeutic targets. We report a patient with an *ETV6::RET*-positive tumor who achieved a complete response to pralsetinib and subsequently developed resistance. Research whole-transcriptome sequencing (WTS) of the resistant lesion identified an expressed in-frame *ETV6::RET* transcript retaining the *RET* kinase domain, with 300 and 196 split reads at the two breakpoints and three discordant mate pairs; research whole-exome sequencing provided limited concordant DNA-level support. A clinically approved targeted DNA-based comprehensive genomic profiling assay applied to material from the same lesion detected *RET* intron 11 and *ETV6* intron 6 rearrangement signals but did not report actionable *ETV6::RET* or *RET* p.L730V. Under the Japanese regulatory framework applicable when selpercatinib was considered, an approved *in vitro* diagnostic or medical device was required to confirm a *RET* fusion for non-lung, non-thyroid solid tumors. Research WTS did not constitute an approved companion diagnostic result, and the approved DNA report did not establish an actionable *ETV6::RET* finding; selpercatinib was therefore not initiated. When DNA profiling yields unresolved rearrangement signals, a clinically validated RNA-based fusion assay and an appropriate regulatory framework for treatment selection may improve access to matched therapy.

## Introduction

Salivary gland secretory carcinoma is a rare malignant salivary gland tumor characterized by recurrent *ETV6* rearrangements. The canonical driver alteration is *ETV6::NTRK3*, which has established the value of molecular testing for this disease (1). In an *ETV6::RET* rearrangement, the *RET* kinase domain is the druggable oncogenic component, whereas *ETV6* contributes the 5-prime fusion segment; this report does not attribute an independent oncogenic role to *ETV6*. A subset of secretory carcinomas lacks *ETV6::NTRK3* and harbors alternative kinase fusions. Identification of the fusion partner is clinically relevant because it may define a therapeutic target.

*ETV6::RET* has been reported as a recurrent noncanonical fusion in salivary gland secretory carcinoma (2). Alternative fusions, including *ETV6::MET* and *VIM::RET*, have been described (3–7). These findings support comprehensive fusion testing in tumors without the canonical *ETV6::NTRK3* rearrangement.

Selective RET inhibitors have demonstrated substantial activity in *RET* fusion-positive cancers. Selpercatinib has shown tumor-agnostic activity across *RET* fusion-positive solid tumors, including cancers other than lung and thyroid cancer (8). It has also demonstrated systemic and intracranial activity in *RET* fusion-positive non-small-cell lung cancer (9). Pralsetinib is active in *RET* fusion-positive non-small cell lung cancer and other *RET* fusion-positive solid tumors (10, 11).

Acquired resistance to selective *RET* inhibitors remains an important clinical issue. In the *RET*gistry Global Consortium, secondary *RET* kinase mutations were identified in a subset of post-progression specimens, although bypass mechanisms such as MET amplification were more frequent (12). *RET* p.L730V was reported as an on-target acquired mutation in plasma cfDNA from patients with *RET* fusion-positive NSCLC who developed resistance to pralsetinib in a conference abstract from the ARROW study (13). L730V/I mutations are located at the roof of the RET solvent-front region and show reduced sensitivity to pralsetinib while retaining relative sensitivity to selpercatinib in preclinical models (14).

We report a patient with *ETV6::RET*-positive salivary gland secretory carcinoma who achieved a complete response to pralsetinib and subsequently developed RET L730V-positive acquired resistance. The central issue was not a binary RNA-positive/DNA-negative result: targeted DNA profiling of the same resistant lesion detected rearrangement signals involving *RET* and *ETV6*, whereas RNA sequencing resolved an expressed actionable *ETV6::RET* fusion that was not generated as a clinically reportable companion diagnostic finding. RET L730V in resistant tissue and plasma provided additional context for acquired resistance.

## Case description

A 49-year-old man presented with a left parotid gland tumor and cervical lymph node metastases. He was diagnosed with salivary gland secretory carcinoma of the left parotid gland, clinically staged as cT2N3bM0. He underwent extended left parotidectomy and left neck dissection of levels I-V. Pathological examination confirmed salivary gland secretory carcinoma. Metastases were present in 50 of 56 ipsilateral lymph nodes: level I, 0/5; level II, 8/8; level III, 4/4; level IV, 12/12; level V, 5/5; and intraparotid lymph nodes, 21/22. Extranodal extension was present in a level II nodal metastasis, which measured 29 x 25 mm. The pathological classification was at least pT3 and pN3b. The surgical margin was focally positive (R1), including the tissue posterior to the mastoid process and a portion of the dissection surface. Re-resection was not performed because it would have required re-entry through the pectoralis major flap reconstruction and was considered excessively invasive. Postoperative radiotherapy (66 Gy in 30 fractions) was selected for local control.

Three months after surgery, computed tomography revealed recurrent lymph node metastases and multiple pulmonary metastases. The PD-L1 combined positive score (22C3) was 4. Pembrolizumab was selected as first-line therapy at the patient’s preference to avoid cytotoxic chemotherapy-associated toxicities, including alopecia. Pembrolizumab was started on October 19, 2022, and administered for two cycles; progressive disease was confirmed by computed tomography on January 6, 2023.

### Diagnostic assessment

After recurrence, archived surgical tumor tissue was analyzed in SCRUM-Japan MONSTAR-SCREEN-2 (UMIN000043899), a nationwide prospective molecular-profiling study for patients with advanced solid tumors that was designed to characterize molecular alterations and support biomarker-guided treatment or trial matching, rather than to evaluate diagnostic accuracy (15). The archived specimen was collected on June 28, 2022, and the MONSTAR-SCREEN-2 test report was issued on November 21, 2022. Caris Life Sciences provided the analysis platform and performed research sequencing, bioinformatics, and variant interpretation at no cost to the study. The study investigators interpreted the findings in the clinical context and made all treatment and trial-eligibility decisions. WES/WTS was performed for research purposes and was not an approved or accredited companion diagnostic in Japan.

### Therapeutic intervention

At another institution, the patient enrolled in cohort K of the Tumor-agnostic Precision Immuno-oncology and Somatic Targeting Rational for You (TAPISTRY) trial (NCT04589845), a global, multicenter, open-label, multi-cohort phase II platform trial evaluating the safety and efficacy of biomarker-matched targeted therapies or immunotherapy in patients with advanced solid tumors. Oral pralsetinib was initiated at 400 mg once daily on February 2, 2023. The metastatic lesions regressed markedly, and the patient’s best overall response was an investigator-assessed complete response according to RECIST version 1.1 (**Figure 1**). Radiographic progressive disease was documented on March 28, 2024, approximately 14 months after pralsetinib initiation.

**Figure 1:**
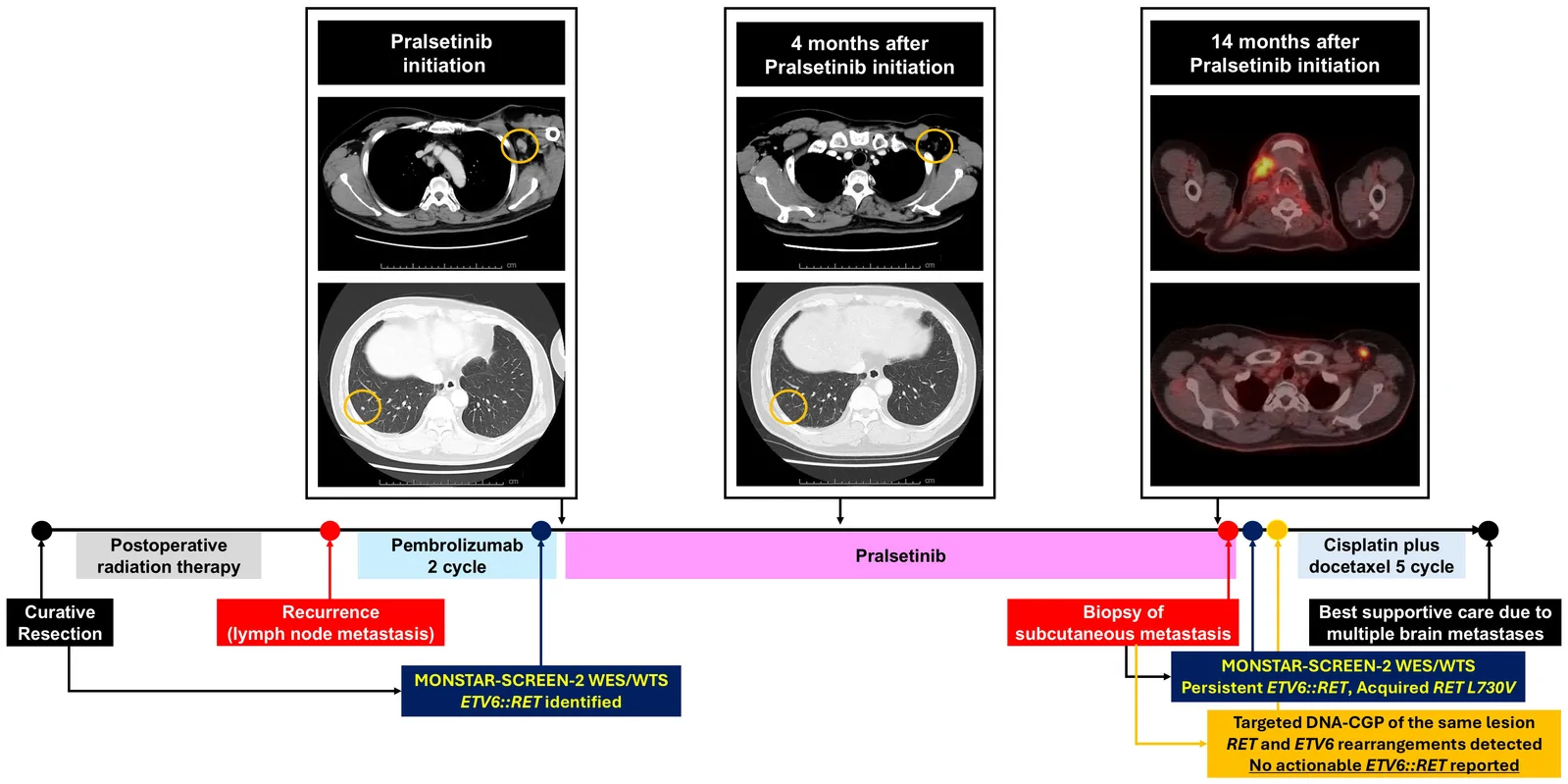
Clinical course and longitudinal molecular findings. Pembrolizumab was administered for two cycles, followed by radiographic progression. Pralsetinib was administered for approximately 14 months until radiographic progression. The surgical specimen showed ETV6::RET by research WES/WTS through SCRUM-Japan MONSTAR-SCREEN-2. Pralsetinib induced an investigator-assessed complete response according to RECIST version 1.1. At progression, the resistant lesion retained ETV6::RET and acquired RET p.L730V. Targeted DNA-based comprehensive genomic profiling of material from the same resistant-lesion tissue block detected RET- and ETV6-related rearrangement signals but did not identify actionable ETV6::RET in the clinically reportable result. WES, whole-exome sequencing; WTS, whole-transcriptome sequencing.

### Follow-up and outcomes

At radiographic progression, a subcutaneous lesion was biopsied as a resistant metastasis. The resistant tissue was analyzed through the same MONSTAR-SCREEN-2 platform. WTS identified an expressed in-frame *ETV6::RET* fusion retaining the *RET* protein kinase domain. The fusion was supported by 300 and 196 split reads at the respective breakpoints and three discordant mate pairs. WES provided limited but concordant DNA-level support for the same rearrangement, with 15 split reads at one breakpoint, no split reads at the reciprocal breakpoint, and no discordant mate pairs (**Figure 2**; **Supplementary Table S1**). *RET* p.L730V was also detected in the resistant lesion. Plasma circulating tumor DNA obtained at progression likewise detected *RET* p.L730V, confirming its presence in both tissue and plasma (**Supplementary Table S2**).

**Figure 2:**
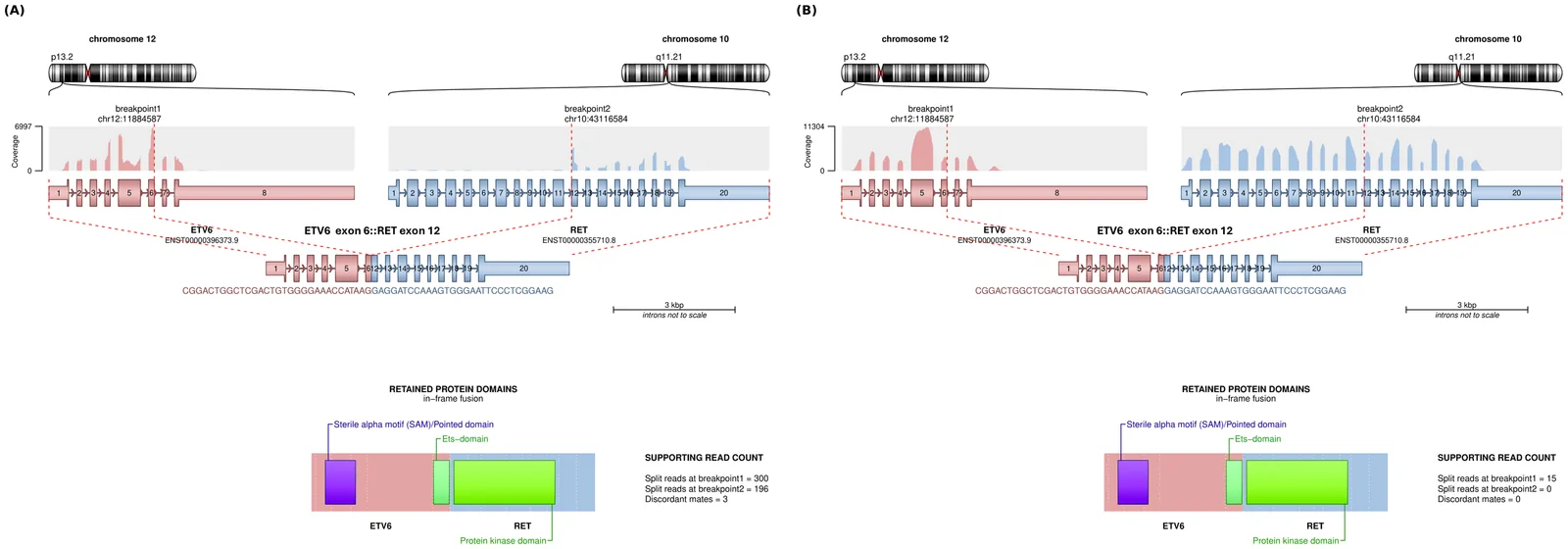
Molecular characterization of the post-pralsetinib resistant lesion. **(A)** Research WTS demonstrated an expressed in-frame *ETV6* exon 6::*RET* exon 12 fusion retaining the *RET* kinase domain, supported by 300 and 196 split reads at the respective breakpoints and three discordant mate pairs. **(B)** Research WES provided limited but concordant DNA-level support for the same rearrangement, with 15 split reads at one breakpoint and no split reads at the reciprocal breakpoint or discordant mate pairs. Breakpoint coordinates in this image are reported on the GRCh38 human reference genome.

To assess eligibility for selpercatinib, material from the same resistant lesion was evaluated using a clinically approved targeted DNA-based comprehensive genomic profiling assay. The companion-diagnostic section did not report an alteration meeting its eligibility criteria. The tumor-profiling section nevertheless reported two *RET* intron 11 rearrangements, including *RET::CSGALNACT2*, and two unpartnered *ETV6* intron 6 rearrangements; it did not report *ETV6::RET* as an actionable fusion or *RET* p.L730V as a reportable short variant (**Table 1**; **Supplementary Table S3**). Research WES/WTS and clinical targeted DNA-CGP used material from the same resistant-lesion tissue block but followed distinct laboratory and analytic workflows. Research profiling reported tumor mutational burden (TMB) 10 mutations/Mb, *TP53* p.L257Q, *RET* p.L730V, *CDKN2A* p.R22fs, and *TERT* promoter c.-124C>T, whereas targeted DNA-CGP reported TMB 0 mutations/Mb, *TP53* p.L194R, *CDKN2A* p.R22fs*4, *ASXL1* p.K568*, two *RET* intron 11 rearrangements, and two *ETV6* intron 6 rearrangements. Differences in capture regions, local coverage, bioinformatic pipelines, variant-calling and reporting thresholds, and TMB algorithms can account for assay-specific outputs even when material is derived from the same tissue block. The results should not be interpreted as a direct comparison of assay accuracy.

**Table 1:** Key targeted DNA-based comprehensive genomic profiling findings.

Category	Finding
Companion-diagnostic interpretation	No alteration was reported as meeting the assay’s companion-diagnostic criteria; tumor-profiling findings are listed below
*RET* rearrangements	Two *RET* intron 11 rearrangements; one was annotated as *RET::CSGALNACT2*
*ETV6* rearrangements	Two *ETV6* intron 6 rearrangements without assigned partners
Actionable fusion report	*ETV6::RET* was not reported as an actionable fusion
RET L730V	Not reported as a reportable short variant
Other genomic alterations	ASXL1 K568*; CDKN2A R22fs*4; TP53 L194R
Microsatellite status	Microsatellite stable
Tumor mutational burden	0 mutations/Mb

The companion-diagnostic interpretation and the tumor-profiling section are separate components of the report. The absence of a qualifying companion-diagnostic result does not mean that no genomic alterations or rearrangement signals were detected.

Selpercatinib was considered because *RET* p.L730V/I has shown greater preclinical resistance to pralsetinib than to selpercatinib (14). Under the Japanese regulatory framework applicable when treatment was considered, the prescribing information for *RET* fusion-positive advanced or recurrent solid tumors other than non-small cell lung cancer and thyroid cancer required confirmation of a *RET* fusion with an approved *in vitro* diagnostic or medical device (16). Research WTS established an expressed in-frame *ETV6::RET* fusion but was not an approved companion diagnostic result, while the approved targeted DNA-CGP report did not identify *ETV6::RET* as the companion-diagnostic finding. Accordingly, the diagnostic criterion for routine on-label care at our institution was not met and selpercatinib was not initiated. Cisplatin plus docetaxel was started on April 19, 2024, and discontinued after the fifth cycle on July 22, 2024. The patient subsequently received palliative radiotherapy to the right cervical lesion beginning on July 29, 2024 (45 Gy in 15 fractions), followed by whole-brain radiotherapy beginning on September 2, 2024 (30 Gy in 10 fractions). He died on September 18, 2024.

### Patient perspective

The patient preferred an initial treatment strategy that avoided cytotoxic chemotherapy-associated toxicities, particularly alopecia. He subsequently agreed to molecular profiling of tumor tissue and plasma. During his lifetime, he signed the SCRUM-Japan MONSTAR-SCREEN-2 consent form, which included consent for publication of his clinical course and genomic findings. After selpercatinib could not be initiated within routine on-label care, he agreed to proceed with cisplatin plus docetaxel. No direct statement from the patient regarding treatment response, disease progression, or inability to receive selpercatinib was documented; therefore, the Patient Perspective is limited to objective information recorded in the medical record.

## Discussion

The principal finding was discordance between research WTS identification of an expressed, kinase-domain-retaining ETV6::RET fusion and clinically reportable targeted DNA-CGP from the same resistant lesion. This was not a binary RNA-positive/DNA-negative result: DNA-CGP detected RET and ETV6 rearrangement signals but did not report actionable ETV6::RET, whereas WTS resolved the expressed in-frame transcript. The assays used different designs and reporting frameworks and were not compared for analytical accuracy.

RET p.L730V should not be regarded as a common resistance mutation. The RETgistry Global Consortium identified RET G810 mutations most frequently among on-target resistance events and also reported V804M, A764G, and L881V; L730V was not recurrent (12). A preliminary ARROW conference abstract identified patient-derived RET p.L730V in plasma after pralsetinib progression (13), whereas Shen et al. provided peer-reviewed evidence that L730V/I reduced sensitivity to pralsetinib more than to selpercatinib (14). The present tissue and plasma findings support an association with the resistant clone but do not establish causality.

Research WES/WTS and clinical DNA-CGP yielded different TMB estimates and variant lists despite using material from the same resistant-lesion tissue block. Differences in capture regions, local coverage, bioinformatic pipelines, variant-calling and reporting thresholds, and TMB algorithms can produce assay-specific outputs. WES mainly interrogates exonic regions, WTS evaluates the expressed fusion transcript, and targeted DNA-CGP applies its own coverage, annotation, and companion-diagnostic rules. Raw clinical-assay data and locus-level coverage metrics were unavailable for reanalysis; therefore, the precise reasons for the discordant TMB estimates and individual variant calls could not be determined. DNA-CGP detected RET intron 11 and ETV6 intron 6 rearrangements but did not report ETV6::RET as actionable. RET::CSGALNACT2 and unpartnered ETV6 events may represent components of a complex rearrangement, but the available data do not permit reconstruction of its architecture. Long-read sequencing, optical genome mapping, or another structural-variant-resolving method would be required.

RNA sequencing can be useful when a kinase fusion is suspected or a DNA assay yields unresolved rearrangement signals. RNA sequencing has identified targetable kinase fusions not resolved by DNA sequencing in other settings (17), and Guha et al. used research transcriptome analysis to identify ETV6::NTRK3 in a rare pediatric secretory breast carcinoma (18). These reports support transcript-level fusion assessment but do not analytically validate MONSTAR-SCREEN-2, establish superiority of research WTS, or justify routine implementation without a clinically validated and appropriately regulated assay.

The reporting discordance affected treatment access. At the time, Japanese prescribing information required RET fusion confirmation with an approved *in vitro* diagnostic or medical device for advanced or recurrent solid tumors other than non-small cell lung cancer and thyroid cancer (16). Research WTS did not meet that regulatory role, and the approved DNA-CGP report did not identify ETV6::RET as the companion-diagnostic finding. Thus, the barrier arose from the labeled diagnostic requirement and reportable assay output, not from absence of DNA rearrangement signals. When DNA profiling yields unresolved signals, a clinically validated RNA-based fusion assay and an appropriate regulatory framework may improve access to matched therapy.

Several limitations apply. The platforms and reporting criteria differed, and the outputs were not directly comparable. No orthogonal structural-variant assay or functional validation of the patient-derived ETV6::RET-p.L730V construct was performed. Selpercatinib was not administered, so this case cannot establish clinical benefit or an altered outcome with that drug. The reason RET p.L730V was absent from the clinically reportable DNA-CGP result remains uncertain, and this single case cannot establish assay performance or a general resistance mechanism. The complete response to pralsetinib and detection of RET p.L730V in resistant tissue and plasma support, but do not prove, the proposed biological interpretation.

## Conclusion

Research WTS identified an expressed *ETV6::RET* fusion, whereas clinically approved targeted DNA-CGP from the same resistant lesion detected related *RET* and *ETV6* rearrangement signals but did not report actionable *ETV6::RET*. The finding concerns complementary assay interpretation and clinical reporting, not analytical superiority of one platform. *RET* p.L730V in resistant tissue and plasma supports a hypothesis of acquired pralsetinib resistance, but a single case without functional validation cannot establish the mechanism or predict benefit from an alternative *RET* inhibitor.

## CARE guidelines statement

This case report was prepared in accordance with the CARE guidelines.

## Data Availability

The data analyzed in this study is subject to the following licenses/restrictions: Deidentified data supporting the findings of this case report are available from the corresponding author upon reasonable request and are subject to applicable ethical and institutional restrictions. Requests to access these datasets should be directed to Shogen Boku, boku.sho@kmu.ac.jp.
