# Supplementary material for: Case Report: Discordance between RNA-identified ETV6::RET and clinically reportable DNA profiling in salivary gland secretory carcinoma
Source: Front Oncol. 2026 Sep 24;16:1967793. doi: 10.3389/fonc.2026.1967793 (PMC13645586; doi:10.3389/fonc.2026.1967793)
Supplement: Supplementary file 1 [file Table1.docx]

***Supplementary Table S1. Research WES/WTS findings in the pralsetinib-resistant lesion.***

| **Category** | **Method / analyte** | **Finding** | **Additional details** |
| --- | --- | --- | --- |
| **Therapy-associated biomarkers** | | | |
| RET fusion | RNA-tumor sequencing | Pathogenic ETV6::RET fusion | ETV6 exon 6::RET exon 12; in-frame. |
| Tumor mutational burden | DNA-tumor sequencing | High, 10 mutations/Mb |  |
| **Genomic signatures** | | | |
| Microsatellite instability | DNA-tumor sequencing | Stable |  |
| Genomic loss of heterozygosity | DNA-tumor sequencing | High | 19% of tested genomic segments exhibited loss of heterozygosity; assay threshold >=16%. |
| **Pathogenic or likely pathogenic alterations** | | | |
| CDKN2A | DNA-tumor sequencing | Pathogenic variant: p.R22fs | Exon 1; c.64delC; variant frequency 58%. |
| RET | RNA-tumor sequencing | Pathogenic fusion: ETV6::RET | ETV6 exon 6::RET exon 12. |
| TERT promoter | DNA-tumor sequencing | Pathogenic variant: c.-124C>T | Variant frequency 62%. |
| TP53 | DNA-tumor sequencing | Likely pathogenic variant: p.L257Q | Exon 7; c.770T>A; variant frequency 56%. |
| **Variants of uncertain significance** | | | |
| KIT | DNA-tumor sequencing | p.V540L | Exon 10; c.1618G>C; variant frequency 75%. |
| RET | DNA-tumor sequencing | p.L730V | Exon 12; c.2188C>G; variant frequency 62%. |
| **Cancer-type relevant negative biomarkers** | | | |
| BRAF | DNA-tumor sequencing | Mutation not detected |  |
| NTRK1/2/3 | RNA-tumor sequencing | Fusion not detected |  |
| EWSR1 | RNA-tumor sequencing | Fusion not detected |  |
| MAML2 | RNA-tumor sequencing | Fusion not detected |  |
| MYB | RNA-tumor sequencing | Fusion not detected |  |
| MYBL1 | RNA-tumor sequencing | Fusion not detected |  |
| AR | RNA-tumor sequencing | Variant transcript not detected |  |
| HRAS | DNA-tumor sequencing | Mutation not detected |  |

*Supplementary Table S2. Plasma ctDNA findings at pralsetinib progression.*

***Assay summary***

| ***Category*** | ***Result*** |
| --- | --- |
| ***Therapy-associated biomarkers*** | *No biomarker result with a therapy association was reported* |
| ***Blood tumor mutational burden*** | *18 mutations/Mb* |
| ***Microsatellite instability*** | *Not detected* |
| ***Tumor fraction*** | *4.3%* |
| ***Incidental germline pathogenic/likely pathogenic variants*** | *None detected* |
| ***Clonal hematopoiesis findings*** | *None detected* |

***Tumor-associated genomic findings***

| ***Gene*** | ***Protein alteration*** | ***DNA alteration*** | ***Variant allele frequency*** | ***Classification*** |
| --- | --- | --- | --- | --- |
| *ATM* | *p.E1783** | *c.5347G>T* | *0.8%* | *Pathogenic variant* |
| *CDKN2A* | *p.R22fs* | *c.64delC* | *3.0%* | *Pathogenic variant* |
| *CREBBP* | *p.I1084fs* | *c.3250delA* | *0.8%* | *Pathogenic variant* |
| *TERT promoter* | *–* | *c.-124C>T* | *4.3%* | *Pathogenic variant* |
| *TP53* | *p.L257Q* | *c.770T>A* | *4.1%* | *Likely pathogenic variant* |
| ***RET*** | ***p.L730V*** | ***c.2188C>G*** | ***3.5%*** | ***Variant of uncertain significance*** |
| *ATR* | *p.E1765K* | *c.5293G>A* | *2.1%* | *Variant of uncertain significance* |
| *SETD2* | *p.R507K* | *c.1520G>A* | *2.5%* | *Variant of uncertain significance* |

***Supplementary Table S3. Clinically reportable targeted DNA-CGP findings from the pralsetinib-resistant lesion.***

| **Parameter** | **Result** |
| --- | --- |
| Quality control | Pass |
| Tumor nuclei | 40% |
| Purity assessment | 23.79% |
| Mean exon depth | 2,144.85× |
| Microsatellite status | Microsatellite stable |
| Tumor mutational burden | 0 mutations/Mb |
| Companion diagnostic interpretation | No alteration was reported as meeting the assay's companion-diagnostic criteria; tumor-profiling findings are listed below. |
| RET p.L730V report status | Not included as a reportable targeted DNA-CGP short-variant result. |

***A. Assay summary***

***B. Rearrangements reported in tumor profiling***

| **Target gene** | **Reported finding** | **Other gene / partner** | **Event type** | **In-frame status** | **Allele fraction, %** | **Supporting read pairs** | **Report status** |
| --- | --- | --- | --- | --- | --- | --- | --- |
| RET | RET intron 11 | No partner assigned | rearrangement | unknown | 13.10 | 162 | likely |
| RET | RET intron 11 | CSGALNACT2 | rearrangement | unknown | 9.28 | 110 | likely |
| ETV6 | ETV6 intron 6 | No partner assigned | truncation | unknown | 7.37 | 150 | likely |
| ETV6 | ETV6 intron 6 | No partner assigned | truncation | unknown | 12.11 | 309 | likely |

***C. Short variants reported in tumor profiling***

| **Gene** | **Protein alteration** | **Functional effect** | **Allele fraction, %** | **Depth** | **Report status** |
| --- | --- | --- | --- | --- | --- |
| NKX2-1 | p.G322S | missense | 41.95 | 298 | unknown |
| CDKN2A | p.R22fs*4 | frameshift | 13.76 | 1025 | likely |
| DOT1L | p.G765S | missense | 50.08 | 1186 | unknown |
| ASXL1 | p.K568* | nonsense | 5.17 | 1295 | likely |
| PTCH1 | p.E44G | missense | 57.51 | 946 | unknown |
| KIT | p.V540L | missense | 47.46 | 1715 | unknown |
| NOTCH2 | p.T89M | missense | 48.99 | 445 | unknown |
| LTK | p.G213_A214insGGG | nonframeshift | 46.79 | 374 | unknown |
| TP53 | p.L194R | missense | 13.62 | 1498 | known |
| PTCH1 | p.S827G | missense | 55.50 | 1337 | unknown |
| PARP1 | p.L698W | missense | 50.99 | 961 | unknown |
| RNF43 | p.R519Q | missense | 46.44 | 1529 | unknown |
| APC | p.S2533C | missense | 48.14 | 2121 | unknown |
| BRCA2 | p.N2436I | missense | 43.34 | 1463 | unknown |
